# Revisiting the Mechanical Work–Energy Framework in Dynamic Biomechanical Systems

**DOI:** 10.3390/bioengineering12090977

**Published:** 2025-09-15

**Authors:** Donglu Shi

**Affiliations:** The Materials Science and Engineering Program, Department of Mechanical and Materials Engineering, Department of Biomedical Engineering, College of Engineering and Applied Science, University of Cincinnati, Cincinnati, OH 45221, USA; donglu.shi@uc.edu

**Keywords:** work–energy relationship, rate of force application, biomechanics, soft matter, muscle contraction, nano delivery, biological tissues, energy partitioning, time-dependent systems

## Abstract

The classical definition of mechanical work, W = F × D, assumes that work depends solely on force magnitude and displacement, independent of loading rate. However, biological tissues exhibit inherent rate sensitivity—muscles demonstrate velocity-dependent force generation governed by Hill’s force–velocity relationship, while connective tissues and joints show load-rate-dependent stiffness and injury thresholds. These rate effects profoundly influence mechanical work, energy dissipation, and functional outcomes. In this work, we revisit the work–energy framework within biomechanics and biomaterials contexts, combining theoretical models, simulations, and a proposed rate-matched nano–bio indentation experiment to quantify how loading rate modulates energy partitioning between recoverable elastic storage and irreversible viscous dissipation. Our analyses span muscle contraction, viscoelastic tissue mechanics, and nanoparticle–membrane interactions, revealing that rapid loading markedly increases viscous dissipation and total mechanical work, even when peak force and displacement remain constant. We demonstrate that classical quasi-static formulations underestimate energy costs and tissue stresses by neglecting temporal dynamics and nonlinear material responses. Our multi-physics experimental–simulation platform bridges this gap, enabling controlled investigation of rate-dependent biomechanical phenomena at the nano–bio interface. These insights inform biomaterials design by emphasizing rate-matching viscoelastic properties to native tissues and guide experimental biomechanics toward capturing full dynamic histories. This unified framework advances understanding of rate-dependent mechanical work, improving predictive modeling, optimizing therapeutic delivery, and enhancing design in sports science, orthopedics, rehabilitation, and nanomedicine.

## 1. Introduction

Mechanical work is a cornerstone of classical physics and engineering, forming the basis for our understanding of energy transfer across a wide range of systems. Traditionally, it is defined as the integral of force over displacement:

(1) W=∫F⋅dx≈F×D

where *F* is the applied force and D the displacement along the force direction [1]. This classical formulation implies that the total mechanical work depends solely on the magnitude of force and displacement, independent of the rate or timing of force application. In biological systems and biomaterials, especially the soft matter, however, this assumption often falls short. Biological tissues—including muscles, tendons, cartilage, and connective tissue—exhibit complex, rate-dependent mechanical behaviors due to their hierarchical, viscoelastic, and nonlinear microstructures [2,3,4]. For example, muscle contraction follows a characteristic force–velocity relationship where rapid shortening reduces force output and affects both mechanical work and metabolic energy consumption [5]. Tendons and ligaments display strain-rate sensitivity that influences stiffness and injury thresholds, critically shaping joint mechanics and movement safety [6,7]. These tissues dissipate energy differently depending on loading rate, reflecting intrinsic viscoelasticity and internal frictional mechanisms not accounted for by classical work definitions [8].

Such rate-dependent phenomena are especially prominent in dynamic activities ranging from rapid muscle contractions in athletic performance to slow, sustained postural control, and are fundamental in rehabilitation science and biomaterial design [9,10]. For instance, high loading rates during impacts or sudden movements can elevate tissue damage risk, whereas controlled loading promotes healing and remodeling [11,12]. Moreover, emerging soft matter engineered to mimic or interface with biological tissues must account for time-dependent mechanical properties to ensure functional integration and durability [13].

Despite their importance, classical mechanics frameworks rarely incorporate the explicit role of force application rate in defining and measuring mechanical work in biological contexts [14,15]. Most biomechanical models simplify work as force–displacement integrals, neglecting the temporal dynamics critical to physiological and pathological processes. This gap limits our understanding of energy transfer, efficiency, and injury mechanisms in living systems.

In this study, we revisit the work–energy relationship with an emphasis on biomechanics and biomaterials, exploring how varying force application rates influence mechanical work, energy partitioning, and functional outcomes in tissues exhibiting viscoelasticity and nonlinear response. Drawing on theoretical analyses, experimental case studies, and simulations, we demonstrate when and why classical work formulations fail in biological systems.

Our approach examines muscle models governed by Hill’s law, viscoelastic tissue behavior via standard mechanical analogs, and frictional energy dissipation in joint interfaces. We further propose experimental methodologies for quantifying rate-dependent mechanical work in biological tissues and biomimetic materials. By integrating classical mechanics with the rich temporal dynamics of biological systems, this work provides a more complete framework for understanding and optimizing biomechanical function, injury prevention, and biomaterial performance.

While prior studies have examined rate effects in isolated systems, no unified framework spanning muscle, tissue, and nano–bio interfaces currently exists—this study addresses that gap through complementary theoretical models, simulations, and proposed experiments.

Classical Definition of Work

In classical mechanics, work W done by a force F acting on a particle as it moves from an initial position x_i_ to x_f_ is defined as

(2) $$W=\int_{x_{i}}^{x_{f}}F\cdot dx$$

For the special case of a constant force acting along the direction of displacement, this integral simplifies to the familiar expression:

(3) W=F×D

where F is the constant magnitude of the force and D is the displacement in the direction of the force [1,2]. This formulation is foundational in physics and engineering and is widely taught in introductory courses and textbooks [2,3,8].

Classical mechanics defines work under a set of simplifying assumptions: the applied force is constant in both magnitude and direction; the displacement occurs in a straight line along the direction of the force; the system is conservative, with no losses to friction, deformation, or thermal dissipation; and the only energy transfer is mechanical, arising from force acting over displacement. Under these idealized conditions, the classical work–energy theorem directly relates work done to the change in kinetic energy:

(4) $$W=\Delta K=\frac{1}{2}mv_{f}^{2}-\frac{1}{2}mv_{i}^{2}$$

where m is the mass of the object, and v_i_ and v_f_ are the initial and final velocities, respectively [1,9]. However, it is important to note that in this classical framework, the duration or rate of force application—whether the force is applied slowly or rapidly—does not appear explicitly in the work equation. The absence of time-dependent terms reflects the Newtonian focus on instantaneous interactions and conservative systems [9,10].

This simplification works well for many physical systems, such as frictionless objects sliding on inclined planes or mass-spring oscillators obeying Hooke’s law. However, in practical and complex systems, energy dissipation, internal deformation, and time-dependent responses often render this formulation insufficient [14]. These discrepancies motivate a deeper inquiry into whether and how the temporal dynamics of force application influence energy transfer, especially in systems exhibiting hysteresis, viscoelasticity, or irreversible processes. Such questions carry both theoretical significance and practical implications—from designing energy-efficient mechanical systems to understanding biological tissue responses—highlighting the need to revisit classical theory in light of modern experimental evidence.

Effect of Rate of Force Application on Mechanical Work

In classical physics, mechanical work is defined as W=∫F⋅dx. This formulation—a cornerstone of Newtonian mechanics—implies that the work done on an object depends solely on the applied force and the displacement along the force direction, with no explicit dependence on time or the rate at which the force is applied [1,2,9]. Consequently, as long as the force-displacement relationship remains constant, classical theory predicts the same amount of work whether the motion occurs over milliseconds or minutes. However, a growing body of experimental evidence across diverse disciplines challenges this assumption of time-independence. In materials science, biomechanics, tribology, and mechanical engineering, the rate at which force is applied critically shapes energy transformation pathways, determining how mechanical work is divided between recoverable (elastic) energy and irreversible (dissipative) losses [4,5,6,12,13].

Rate-dependent effects manifest in several key ways:

Inelastic and Microstructural Responses: Rapid force application often triggers inelastic deformation, microcracking, and local heating, particularly in polymers, composites, metals, and biological tissues. These phenomena dissipate mechanical energy as heat or internal damage, reducing recoverable work [3,5,6].

Wave Propagation and Shock Loading: At high strain rates, materials experience complex dynamic responses that significantly alter energy dissipation mechanisms. Stress waves and shock fronts propagate through the material, exciting transient vibrational modes and inertial effects that are not accounted for in classical work calculations [11,12]. These additional pathways can lead to increased energy loss, localized heating, and microstructural deformation, highlighting the need for rate-sensitive models when analyzing fast-loading scenarios in biological tissues, polymers, and engineered materials.

Relaxation and Recovery at Low Rates: Slow loading allows sufficient time for molecular or microstructural relaxation and energy redistribution within the material, thereby reducing hysteresis and damping losses commonly observed in viscoelastic and thermally sensitive systems [4,5]. This leads to a greater fraction of the input work being stored reversibly and a smaller fraction dissipated irreversibly.

These observations raise a fundamental theoretical question with direct practical implications: Is the classical work expression, W = ∫F⋅dx, truly complete for describing mechanical work in real, rate-dependent systems? Or does the temporal profile of force application fundamentally alter the effective work done and its partitioning between stored and dissipated energy?

Biomechanics:

Muscle-Tendon Units

Muscle-tendon units exhibit velocity-dependent force generation and associated metabolic costs, making contraction speed a critical factor for energy efficiency and injury risk [6]. Faster contractions reduce force output per cycle while increasing the metabolic demand, and tendon elasticity further modulates force transmission and energy storage. Understanding these dynamics requires integrating rate-dependent biomechanical models that account for both muscle and tendon properties.

Polymer Mechanics

Polymeric and soft biomaterials often display time-temperature superposition and strain-rate sensitive modulus effects, which classical elasticity models cannot fully capture [3,5]. Accurate prediction of their behavior under varying loading rates requires constitutive models that account for viscoelasticity, microstructural relaxation, and thermal effects. Such models are essential for designing biomimetic materials and interpreting experimental results in biomechanics and tissue engineering.

Frictional Interfaces

Frictional contacts in biological and engineered systems exhibit stick-slip phenomena, velocity-dependent friction, and rate-sensitive heat generation, all of which directly influence energy dissipation and system stability [12]. These effects can dominate local energy budgets in joints, nanoscale contacts, and mechanical interfaces, emphasizing the need to consider dynamic and rate-dependent friction in both experimental analysis and modeling.

Engineering Design: Dynamic loads applied over short timescales can cause failure modes and fatigue damage undetectable by static or quasi-static analyses, necessitating rate-aware design criteria [13,14].

Even within the relativistic regime, the explicit inclusion of time and frame-dependence in energy formulations via Lorentz transformations emphasizes that rate and temporal context are inseparable from an accurate energy description [10]. The contrast between the simplicity of classical work integrals and the complexity of real-world, rate-sensitive materials highlights the need to reconsider how time-dependent forces influence mechanical work.

The microscopic mechanisms of energy dissipation underlie the observed rate-dependent effects in mechanical work. These include the roles of viscoelasticity, plasticity, friction, and biological processes in modulating energy transfer. This highlights the practical need for generalized or extended work–energy frameworks that incorporate temporal dynamics and material-specific constitutive behavior. Understanding how classical mechanics intersects with time-dependent and rate-sensitive behaviors in real materials provides the foundation for analyzing energy partitioning and dissipation in biological tissues and biomaterials.

Classical Work–Energy Theorem

In idealized conservative systems, the work–energy theorem remains the cornerstone for relating force to energy. Equation (4) asserts that the work done by a net force equals the change in kinetic energy of a particle of mass m, independent of the time duration over which the force is applied [1,2,9]. While time does not explicitly appear in the total work expression, it enters indirectly through power, defined as the rate at which work is performed:

(5) P=F⋅v

where p is power, F is force, and v is velocity. Total work can then be expressed as

(6) $$W=\int_{0}^{t}P\;dt=\int_{0}^{t}F\cdot v\;dt$$

However, the classical theorem rests on several key assumptions. (1) Forces are applied either instantaneously or sufficiently slowly (quasi-statically), such that inertial and dynamic effects are negligible. (2) The system exhibits no rate dependence or memory effects and behaves as an ideal elastic body. (3) Internal state variables—such as temperature changes, molecular relaxation, or deformation history—do not influence the mechanical response. (4) Energy losses via internal friction, plastic deformation, or other irreversible processes are absent.

When these assumptions are violated, as frequently occurs in real materials and complex systems, the clear-cut separation between force, displacement, and time breaks down. For example, in viscoelastic materials, the stress depends on both strain and strain rate, introducing hysteresis and energy dissipation that cannot be captured by purely elastic models. Similarly, plastic deformation involves irreversible structural changes dependent on loading rate, and frictional interfaces exhibit velocity-dependent forces that convert mechanical energy into heat or sound.

Under such conditions, the classical work–energy theorem remains mathematically valid but insufficient for capturing the complete energy transfer and dissipation mechanisms. The total mechanical work input no longer equals solely recoverable kinetic or potential energy changes but also includes rate-dependent dissipative losses distributed across microscopic degrees of freedom. Consequently, a more nuanced framework that incorporates temporal dynamics, material constitutive behavior, and internal state evolution is necessary to fully describe mechanical work in realistic systems.

While these classical and relativistic frameworks provide a rigorous basis for understanding mechanical work and energy in idealized systems, biological tissues and biomaterials frequently violate these assumptions due to their viscoelasticity, complex hierarchical structure, and active physiological processes. These characteristics introduce pronounced rate-dependent and dissipative behaviors that require extended theoretical treatment beyond the classical work–energy theorem.

Relativistic Corrections

Although, in practice, humans and even mechanical machines do not move at velocities approaching the speed of light, we include a brief discussion to provide a complete theoretical background in the introduction. At such extreme velocities, classical mechanics fails to accurately describe particle dynamics. In this regime, relativistic mechanics, governed by Einstein’s theory of Special Relativity, becomes the appropriate framework. The relativistic kinetic energy K_E_ is given by

(7) $$K_{E}=\left(\gamma -1\right)mc^{2}$$

where the Lorentz factor γ is

(8) $$\gamma =\frac{1}{\sqrt{1-\frac{v^{2}}{c^{2}}}}$$

In the low-velocity limit (v ≪ c), this expression reduces to the classical kinetic energy, $\frac{1}{2}mv^{2}$. However, as the velocity approaches the speed of light (v → c), the required energy increases sharply, ultimately becoming infinite to accelerate a mass to c [7,10,15]. The classical work–energy theorem relies on several key assumptions: (1) Forces are applied instantaneously or sufficiently slowly (quasi-statically), such that inertial and dynamic effects are negligible. (2) The system exhibits no rate dependence or memory effects, behaving as an ideal elastic body. (3) Internal state variables—such as temperature changes, molecular relaxation, or deformation history—do not influence the mechanical response. (4) Energy losses via internal friction, plastic deformation, or other irreversible processes are absent.

Relativistic dynamics also alters the relationship between force and acceleration. Specifically, as velocity increases, the effective inertia (or relativistic mass) grows, causing acceleration to diminish under constant force. The relativistic form of Newton’s second law becomes

(9) $$F=\frac{d}{dt}\left(\gamma mv\right)$$

This dependence introduces velocity into the force equation, which in turn affects the integration of work and energy. Nonetheless, the temporal profile of force application—whether the force is applied slowly or rapidly—does not fundamentally alter the total energy predicted by relativistic theory. Rather, the modifications to kinetic energy arise from the velocity-dependent geometry of spacetime and not from rate-of-application effects per se [7,10].

Thus, while relativistic mechanics provides significant corrections to classical kinetic energy and force interpretations at high velocities, it does not generalize the classical work–energy theorem to encompass rate-dependent material behaviors or dissipative processes. It presupposes idealized, conservative systems without friction or internal energy loss. This contrast highlights the essential need for separate theoretical treatment when addressing rate effects in real materials—where internal dissipation, hysteresis, and nonlinear responses dominate energy transformations.

Energy Dissipation in Biological Systems and Biomaterials

Unlike the idealized assumptions of classical or relativistic mechanics, biological tissues and biomaterials are inherently non-conservative systems exhibiting complex, rate-dependent behaviors where mechanical energy is rarely fully conserved. Instead, the energy input into these systems partitions into multiple pathways involving irreversible dissipation driven by microstructural dynamics, fluid flow, biochemical processes, and thermomechanical interactions. The rate at which forces are applied profoundly influences how energy is stored, transformed, and lost, shaping physiological function, tissue adaptation, and injury risk.

Several key microscopic and mesoscopic mechanisms underlie these rate-dependent responses in biological and biomimetic materials:

Viscoelasticity and Tissue Microstructure: Biological tissues such as muscle, tendon, cartilage, and skin exhibit viscoelastic behavior where stress depends on both instantaneous strain and strain rate. This reflects the hierarchical organization of collagen fibers, extracellular matrix components, and interstitial fluids, producing time-dependent deformation and energy dissipation through internal friction, molecular rearrangement, and fluid flow within porous networks. Constitutive models like Maxwell, Kelvin–Voigt, and Standard Linear Solid analogs effectively describe these behaviors, capturing hysteresis loops observed in cyclic loading that directly relate to dissipated energy [3,4,5,16]. Such viscoelastic dissipation plays a critical role in protecting tissues from damage under dynamic loading and enables energy recycling during locomotion.

Many real materials—such as polymers, biological tissues, and damping composites exhibit viscoelastic behavior, in which force depends on both displacement and velocity. A simple representation is the Kelvin–Voigt model [5]:

(10) F=kx+ηdxdt

where η is the damping coefficient. The total work done becomes

(11) $$W=\int Fdx=\int \left(kx+\eta x^{˙}\right)dx$$

The first term represents elastic storage, while the second corresponds to viscous dissipation. Faster displacement ($x^{˙}$ large) increases viscous losses, requiring greater total energy input [6,7]. This rate-dependence is critical in impact testing, where high strain rates significantly increase measured stiffness and damping [8].

Rate-Dependent Plasticity and Remodeling: Although classical plasticity models apply mainly to metals and polymers, biological tissues undergo irreversible microstructural changes—such as microtears, collagen crosslinking, or cellular remodeling—that depend strongly on loading rate and magnitude. For example, tendon and ligament failure thresholds increase under rapid loading, a phenomenon akin to strain rate hardening seen in synthetic materials. These rate-dependent plastic and remodeling processes govern tissue durability, healing, and adaptation to mechanical stimuli, underscoring the need for constitutive laws that incorporate biological time scales and mechanotransduction pathways [7,13,17].

### Muscle

Biological muscles display velocity-dependent force generation, as described by Hill’s equation [13]:

(12) $$\left(F+a\right)\left(v+b\right)=\left(Fmax+a\right)b$$

where F is the muscle force, v is the shortening velocity, and a and b are empirical constants. While these classical and relativistic frameworks provide a rigorous basis for understanding mechanical work and energy in idealized systems, biological tissues and biomaterials frequently violate these assumptions due to their viscoelasticity, complex hierarchical structure, and active physiological processes. These characteristics introduce pronounced rate-dependent and dissipative behaviors that require extended theoretical treatment beyond the classical work–energy theorem. In muscle mechanics, for example, the force–velocity relationship described by Hill’s law demonstrates that faster contractions produce less force, thereby reducing the mechanical work per cycle and altering the metabolic cost [14,15]. Conversely, slow, sustained contractions produce higher force but incur greater energy cost due to prolonged muscle activation. Rapid contractions, while metabolically efficient per unit time, may decrease mechanical efficiency, increase injury risk, and rely more heavily on elastic energy storage in tendons. Slow contractions, in contrast, generate higher total force but demand greater metabolic expenditure. This rate-dependence is central to sports biomechanics (e.g., optimizing sprint vs. endurance performance), rehabilitation engineering (e.g., selecting contraction speeds for safe and effective strengthening), and injury prevention (e.g., avoiding excessive eccentric loading during rapid movements) [6,7,13,14,15].

These mechanisms necessitate extending the classical work–energy framework to explicitly consider rate-dependent energy partitioning. In biological contexts, the total mechanical work W can be expressed as

W = W_stored_ + W_dissipated_ (13)

where W_stored_ represents recoverable elastic energy stored in tissues and biomaterials, and W_dissipated_ encompasses irreversible losses due to viscoelastic damping, plastic remodeling, frictional heating, fluid flow, and biochemical energy conversion. The proportions of stored versus dissipated energy are highly sensitive to the loading rate. For instance, a tendon stretched slowly allows molecular relaxation and fluid redistribution, minimizing dissipation, whereas rapid loading traps fluid and strains collagen networks, increasing energy loss and injury risk [5,6]. Similarly, skeletal muscle exhibits a well-characterized force-velocity relationship where rapid contractions reduce force output and metabolic efficiency, profoundly influencing mechanical work generation and physiological performance [6,18,19].

These observations highlight a vital conclusion: while the classical expression W = ∫F⋅dx elegantly defines total mechanical energy transfer, it is incomplete for biological and biomimetic materials where internal dissipation, nonlinear mechanics, and rate effects are prominent. A more comprehensive energy balance must incorporate material-specific constitutive laws, temporal dynamics, and the complex partitioning of energy into reversible and irreversible components [4,7,14].

It is important to emphasize that the classical work definition W = F × D remains valid in the non-relativistic regime when interpreted as the total mechanical energy transferred along a displacement path. Apparent discrepancies in energy conservation arise when experiments or models capture only partial energy pathways—such as kinetic energy or elastic strain energy—while neglecting microscale dissipative processes like heat generation, fluid flow, acoustic emissions, or biochemical energy transformations. When all relevant energy channels are accounted for, the total work matches the sum of all produced energy forms, regardless of loading rate. Thus, the critical role of force application rate lies not in violating the fundamental work equation but in governing how mechanical work partitions between recoverable storage and dissipative loss in biological and biomimetic systems.

Internal microstructural dynamics, phase transformations, thermal effects, and other irreversible processes cause a portion of the mechanical work input to be dissipated rather than fully recoverable. This results in energy losses that depend strongly on how quickly forces are applied. The following are some of the typical systems with recoverable elastic energy and irreversible losses.

Force Rate in Biomechanical and Soft Matter Systems

When force is applied rapidly in complex materials—particularly in biological tissues and biomaterials (soft matter)—several energy loss mechanisms become especially significant. By integrating classical mechanics with rate-sensitive constitutive laws and time-dependent dynamics, we can build a predictive framework for mechanical work that reflects how biological tissues and biomaterials actually behave. The temporal profile of force—gradual rise, abrupt spike, oscillation, or sustained load—strongly influences viscoelastic damping, poroelastic flow, frictional heating, microdamage, and active metabolic processes.

Table 1 presents a selection of representative dynamic systems across biomechanics and related fields, illustrating how the rate of force application critically influences mechanical responses and energy transfer. Biological tissues, such as muscle and tendon, exhibit pronounced rate-dependent behaviors characterized by viscoelasticity and force-velocity relationships that significantly affect functional outcomes and injury risk [2,4,5]. For example, skeletal muscle contractions follow Hill’s law, where increasing contraction velocity reduces force output and mechanical work per cycle, impacting metabolic cost and performance efficiency [4,8]. Similarly, connective tissues like tendons and cartilage demonstrate strain-rate dependent stiffness and damping properties, which are essential for load bearing and shock absorption during dynamic activities [6,11,14]. These biomechanical systems operate within velocity regimes far below relativistic speeds but require constitutive models that account for temporal dynamics and dissipative mechanisms to accurately predict energy partitioning [9,10,15].

## 2. Theoretical and Simulation Results

### 2.1. Simulating Rate-Dependent Energy Partitioning in Biomechanical Systems

Viscoelastic Tissue and Muscle Contraction Dynamics

We conducted simulations under two representative biomechanical conditions to investigate rate-dependent energy partitioning. First, we examined how viscous dissipation scales with loading rate in a Kelvin–Voigt bone/tendon analogue, quantifying the trade-off between recoverable elastic storage and irreversible viscous loss. Second, we analyzed how the mechanical work performed by muscle during a single contraction changes with shortening velocity, employing a Hill-type force–velocity relation to capture the underlying physiological constraints.

Kelvin–Voigt tissue model—slow vs. fast stretch

Model: $F\left(t\right)=k\;x\left(t\right)+\eta \;x^{˙}\left(t\right)$ (Kelvin–Voigt)

where

- F(t) = force at time t (N);
- k = elastic stiffness (N/m);
- η = viscous damping coefficient (N·s/m);
- x(t) = displacement at time t (m);
- x˙(t) = velocity (dx/dt) (m/s);
- D = total imposed displacement (m);
- T = loading duration (s);
- W_elastic_ = elastic (recoverable) work (J);
- W_visc_ = viscous (dissipated) work (J);
- W_total_ = total input work (J).

Representative parameter values (tendon-like specimen) are

k = 1000 N/m, η = 50 N·s/m, D = 0.10 m.

The detailed steps of these calculations and the full derivations are provided in Appendix A, while only the key governing equations are summarized here for clarity.

Under the Hill relation, faster shortening reduces force and reduces mechanical work per contraction (2.25 J vs. 4.083 J here). Metabolic cost per unit time, muscle activation dynamics, and tendon elastic recycling will further modulate efficiency—but the pure mechanical work falls with increased contraction speed.

Table 2 summarizes characteristic force rise times, accelerations, and normalized velocity fractions (v/c) for a variety of biomechanical and bioengineering systems, illustrating the broad spectrum of temporal scales over which force is applied in biological contexts. These parameters are critical for understanding how rate-dependent effects influence mechanical work and energy dissipation in soft tissues, muscle contractions, and other biomaterials [2,4,6,10]. For example, typical human muscle contractions occur over tens of milliseconds with accelerations on the order of 10 m/s^2^, while more rapid loading scenarios—such as impacts or high-intensity athletic maneuvers—can exhibit much shorter rise times and higher accelerations, profoundly affecting tissue mechanics and metabolic cost [7,8,14]. This table provides a quantitative framework for relating mechanical loading rates to physiological function, injury risk, and material behavior in biological systems.

**Figure 1:**
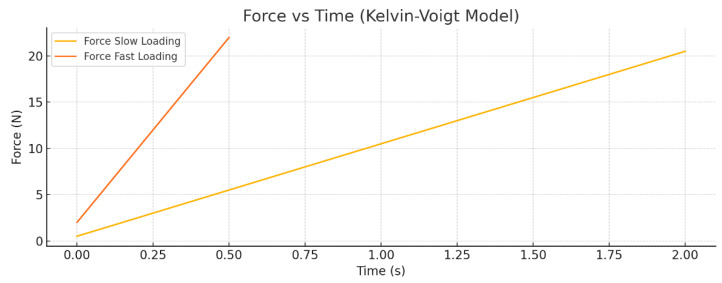
Force versus time for the Kelvin–Voigt viscoelastic model under slow (2 s) and fast (0.5 s) linear displacement ramps from 0 to 0.02 m. The fast loading exhibits a higher peak force and a quicker force rise due to viscous effects.

Figure 1 shows the Force versus Time response of the Kelvin–Voigt viscoelastic model subjected to a linear displacement ramp from 0 to 0.02 m. Two loading rates are compared: slow loading over 2 s and fast loading over 0.5 s. The plot clearly illustrates that the peak force is significantly greater during the fast loading due to the increased contribution of the viscous damping element. Additionally, the force rises more rapidly for the fast-loading case, reflecting the rate-dependent nature of the viscoelastic material. The slower loading results in a lower peak force and a more gradual force increase.

**Figure 2:**
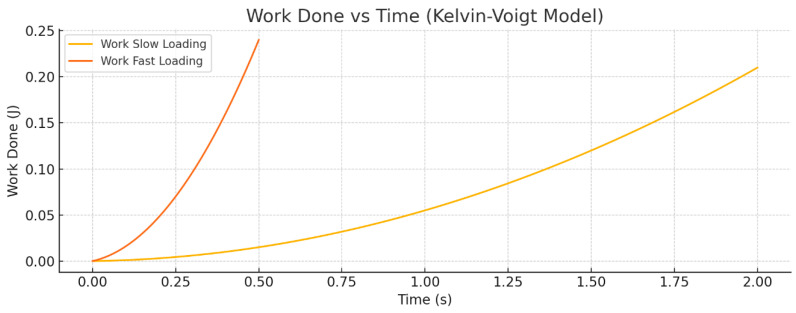
Work done versus time for the Kelvin–Voigt model under slow and fast displacement ramps. The fast-loading results in increased total work, reflecting greater energy dissipation from viscous damping.

Figure 2 presents the Work done versus Time curves for the same loading scenarios. The total mechanical work performed on the system increases over time as the displacement ramps up. The fast-loading condition results in a steeper rise in work done, indicating higher energy dissipation due to viscous losses. This greater cumulative work under fast loading demonstrates the rate sensitivity and energy dissipation characteristic of the Kelvin–Voigt model, where faster deformation leads to more work being converted into heat by the damping element compared to slow loading.

### 2.2. Rate-Dependent Nanoparticle Entry Through Cell Membranes: A Viscoelastic Indentation Model

Having examined tissue- and muscle-scale rate-dependent behaviors, we now turn to the nanoscale, where similar mechanical principles govern the interaction between therapeutic nanoparticles and cellular membranes. Understanding how entry dynamics depend on force application rate is critical for predicting endocytosis efficiency, membrane rupture thresholds, and overall biocompatibility. In this subsection, we model the process of a single nanoparticle approaching and indenting a viscoelastic cell membrane using a Kelvin–Voigt framework with optional adhesion. This provides a simplified but tractable representation of the balance between elastic storage, viscous dissipation, and adhesive interactions during particle uptake. We first establish the governing equations, define the relevant mechanical variables, and then analyze how variations in loading rate influence peak forces and energy partitioning.

**Figure 3:**
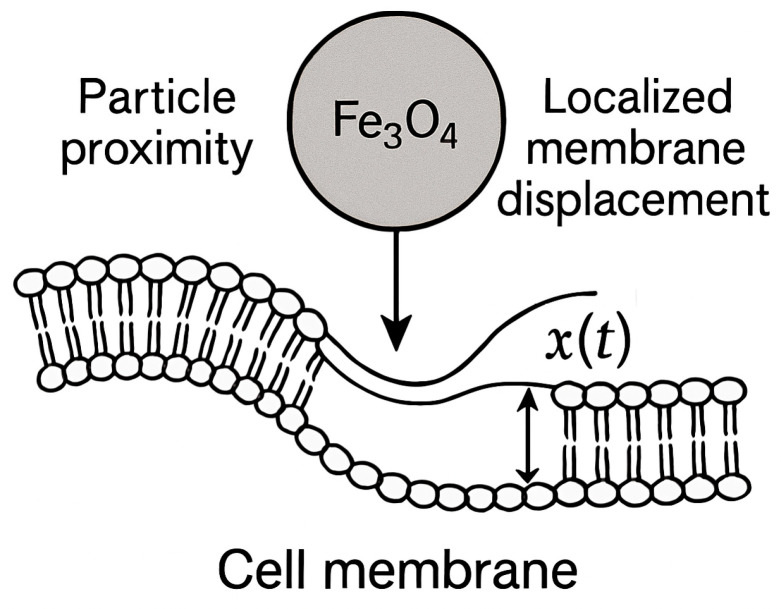
Schematic diagram showing that a single nanoparticle (e.g., Fe_3_O_4_) approaches and indents a cell membrane, applying a localized displacement x(t) until the membrane either encloses the particle (endocytosis) or ruptures.

A single nanoparticle (e.g., Fe_3_O_4_) approaches and indents a cell membrane, applying a localized displacement x(t) until the membrane either encloses the particle (endocytosis) or ruptures (Figure 3). The membrane + cortex is modeled as an effective viscoelastic shell (linear Kelvin–Voigt element for first order insight) with an adhesive contribution. Rate of entry (particle velocity) controls x˙ and therefore viscous dissipation; different rates produce different peak forces and total work (energy dissipated + stored).

Take the local contact/indentation coordinate x(t) (0 → D is required indentation to engulf/penetrate). Use a Kelvin–Voigt form with an adhesion term $F_{adh}\left(x\right)$ if needed:

(14) $$F\left(t\right)=kx\left(t\right)+\eta x^{˙}\left(t\right)+F_{adh}\left(x\right)$$

Definitions of variables used in this model:

- F(t): total force on the membrane at time t (N);
- k: effective elastic stiffness of membrane + cortex (N/m);
- η: viscous damping coefficient (N·s/m);
- x(t): indentation displacement at time t (m);
- x˙(t): indentation velocity (m/s);
- D: required indentation depth for engulfment or penetration (m);
- T: loading duration or indentation time (s);
- F_adh(_x_)_: adhesion force contribution (N), possibly distance-dependent;
- W_el_: elastic (recoverable) work (J);
- W_visc_: viscous (dissipated) work (J);
- W_total_: total input work (J).

Total mechanical work done on the membrane while moving from x = 0 to x = D is

(15) $$W_{total}=\int_{0}^{D}F\;dx=\int_{0}^{D}kx\;dx+\int_{0}^{T}\eta \dot{x^{2}}\;dt+\int_{0}^{D}F_{adh}\left(x\right)\;dx$$

The elastic (recoverable) part:

(16) $$W_{el}=\frac{1}{2}kD^{2}$$

The viscous (dissipated) part reduces to a simple scaling for a linear ramp x(t) = D(t/T):

(17) $$W_{visc}=\eta \int_{0}^{T}\dot{x^{2}}\;dt=\eta \int_{0}^{T}{\left(\frac{D}{T}\right)}^{2}dt=\eta \frac{D^{2}}{T}$$

This last expression is the same scaling used in the Kelvin–Voigt example and highlights how viscous losses grow as loading time T decreases. Adhesion and membrane bending (Helfrich) can be included if considering more realism: add an adhesion energy term or an effective pull-off force (distance dependent). Bending/tension contributions give a non-linear k(x) (or effective spring constant depending on contact radius). Those can be included numerically.

Representative numeric example

Required indentation, D:

D = 100 nm = 1.00 × 10^−7^ m.

Effective stiffness, k:

k = 0.010 N/m

Effective viscous coefficient, η:

η = 1.0 × 10^−6^ N s/m.

Compute elastic stored energy, $W_{el}$:

$$D^{2}={\left(1.00\times 10^{-7}\right)}^{2}=1.00\times 10^{-14}\;m^{2}$$

Calculation:

$$W_{el}=\frac{1}{2}kD^{2}$$

Step 1. Multiply the prefactor

$\frac{1}{2}$ × k = 0.5 × 0.010 = 0.005

Step 2. Multiply by $D^{2}$

0.005 × 1.00 × 10 − 14 = 5.00 × 10^−17^ J

Result

$$W_{el}=5.00\times 10^{-17}\;J$$

Now viscous dissipation for two entry times:

$$\mathrm{Slow}\;\mathrm{entry}:\;T=0.10\;s\;\left(v=D/T=\frac{1.00\times 10^{-7}}{0.10}=1.00\times 10^{-6}\;m/s\right)$$

$$W_{\mathrm{visc},\mathrm{slow}}=\frac{\eta D^{2}}{T}=\frac{\left(1.0\times 10^{-6}\right)\times \left(1.00\times 10^{-14}\right)}{0.10}$$

$$\mathrm{Numerator}:\;\left(1.0\times 10^{-6}\right)\times \left(1.00\times 10^{-14}\right)=1.00\times 10^{-20}$$

$$\mathrm{Divide}\;\mathrm{by}\;0.10:\;\frac{1.00\times 10^{-20}}{0.10}=1.00\times 10^{-19}\;J$$

$$\mathrm{Fast}\;\mathrm{entry}:\;T=1.00\times 10^{-4}\;s\;\left(v=1.00\times 10^{-3}\;m/s\right)$$

$$W_{\mathrm{visc},\mathrm{fast}}=\frac{\left(1.0\times 10^{-6}\right)\times \left(1.00\times 10^{-14}\right)}{1.00\times 10^{-4}}$$

$$\mathrm{Numerator}\;\mathrm{same}:\;1.00\times 10^{-20}$$

$$\mathrm{Divide}\;\mathrm{by}\;1.00\times 10^{-4}:\;\frac{1.00\times 10^{-20}}{1.00\times 10^{-4}}=1.00\times 10^{-16}\;J$$

Compare elastic vs. viscous:

$$W_{\mathrm{el}}=5.00\times 10^{-17}\;J.$$

$$W_{\mathrm{visc},\mathrm{slow}}=1.00\times 10^{-19}\;J\;\left(\mathrm{two}\;\mathrm{orders}\;\mathrm{of}\;\mathrm{magnitude}\;\mathrm{smaller}\;\mathrm{than}\;\mathrm{elastic}\right).$$

$$W_{\mathrm{visc},\mathrm{fast}}=1.00\times 10^{-16}\;J\approx 2\times W_{\mathrm{el}}\;\left(\mathrm{viscous}\;\mathrm{dissipation}\;\mathrm{becomes}\;\mathrm{comparable}\;\mathrm{or}\;\mathrm{dominant}\right).$$

For the parameters used in this study—indentation depth D = 100 nm, effective stiffness k = 0.010 N/m, and effective viscous coefficient η = 1.0 × 10^−6^  N—reducing the indentation time from T = 0.10 s to T = 1 × 10^−4^ s increases viscous dissipation by a factor of 10^3^ and shifts the system from an elastic-dominated to a viscous-dominated energy budget. This change affects peak forces and alters the energy available for membrane remodeling versus heating or damage.

**Figure 4:**
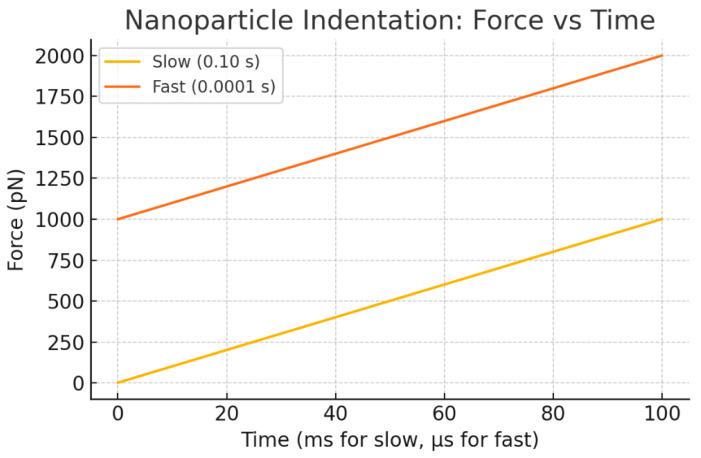
**Force–time responses of a nanoparticle transported through a viscoelastic membrane modeled by a Kelvin–Voigt system**. The plots compare slow and fast force ramp rates, illustrating the rate-dependent viscous dissipation. Faster loading results in significantly higher viscous work compared to elastic energy, implying important considerations for nanoparticle size, surface chemistry, and delivery speed to balance effective transport and minimize membrane damage or heating.

Figure 4 presents the dynamic mechanical response of nanoparticle (NP) transport through a viscoelastic membrane, modeled here by a Kelvin–Voigt system. The force applied to the NP as a function of time (force–time plot) and the cumulative mechanical work done (work–time plot) are shown for two loading scenarios: slow and fast ramp-up of force. The underlying analytic model treats the membrane as a viscoelastic medium characterized by an effective viscosity η and elastic modulus E. The total mechanical work W required to drive NP displacement D over time T comprises elastic and viscous components.

The viscous work scales as

$$W_{\mathrm{visc}}=\eta \frac{D^{2}}{T}$$

where η is the viscous damping coefficient, D is the characteristic NP displacement, and T is the characteristic timescale of force application.

**Figure 5:**
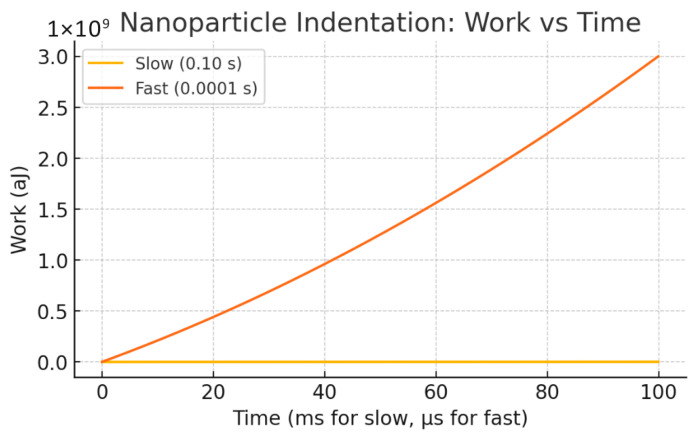
Work versus time curves for nanoparticle transport through a viscoelastic membrane under slow and fast force ramps modeled by a Kelvin–Voigt system. The faster ramp leads to a steeper increase and higher total mechanical work due to enhanced viscous dissipation, whereas the slower ramp shows predominantly elastic work accumulation over a longer time. This illustrates the significant effect of loading rate on energy dissipation during nanoparticle delivery.

Figure 5 depicts the temporal evolution of mechanical work performed during nanoparticle transport through the viscoelastic membrane under different force application rates. The work–time curves reveal how the total mechanical energy input accumulates over time for both slow and fast loading ramps. As expected from the Kelvin–Voigt viscoelastic model, the fast ramp induces a rapid rise in work due to increased viscous dissipation, while the slow ramp shows a more gradual energy increase dominated by elastic contributions. This dynamic highlights the critical influence of loading rate on the mechanical energy landscape experienced by the nanoparticle and has direct implications for optimizing delivery protocols that balance efficiency with membrane integrity.

Figure 4 and Figure 5 together provide a comprehensive view of the mechanical energy dynamics involved in nanoparticle (NP) transport through a viscoelastic membrane modeled by a Kelvin–Voigt system. Figure 4 illustrates how the applied force evolves differently under slow and fast ramp conditions, revealing a pronounced rate-dependent response. The fast ramp generates a sharp force increase due to the viscous resistance of the membrane, whereas the slow ramp produces a gentler, more gradual force rise dominated by elastic deformation.

Analysis of the mechanical energy (Figure 5) shows that faster loading results in significantly higher viscous work compared to elastic energy. This finding highlights important considerations for nanoparticle size, surface chemistry, and delivery speed, which must be balanced to optimize transport efficiency while minimizing potential membrane damage or heating.

These results build on the representative numerical example described above, where a single nanoparticle indents a viscoelastic membrane modeled as a Kelvin–Voigt element. The chosen parameters—membrane stiffness k, viscous damping η, indentation depth D, and loading durations T—illustrate the contrasting effects of slow versus fast entry. Figure 4 and Figure 5 show the corresponding force–time responses and cumulative work, highlighting how faster indentation increases viscous dissipation and total mechanical work, while slower indentation is dominated by elastic energy accumulation. These dynamics explicitly link the analytical expressions for W_elastic_ and W_visc_ to time-dependent mechanical behavior, demonstrating the critical role of loading rate in energy partitioning.

The results also indicate that nanoparticle size, surface chemistry, and delivery speed strongly influence viscous dissipation and mechanical stress. Faster delivery increases the risk of membrane damage or localized heating, emphasizing the need to optimize loading rates, NP characteristics, and membrane mechanics for safe and efficient nanoparticle transport.

### 2.3. Rate-Matched Nano–Bio Indentation: A Unified Experimental Framework for Force-Rate Dependent Energy Partitioning at the Nano–Bio Interface

A key gap in the current literature on time-dependent biomechanical systems is the lack of a unified experimental framework that directly quantifies the role of force application rate in the partitioning of work into stored versus dissipated energy at the nano–bio interface. Existing studies have explored related phenomena in isolation: nanoindentation has been used to characterize biological materials at small scales [17], poroelastic and viscoelastic indentation responses of hydrated tissues and hydrogels have been investigated [18,19,20,21,22,23], and rate-dependent adhesive and frictional effects at soft interfaces have been probed via atomic force microscopy [21]. While these works have provided valuable insight into elastic, viscous, and poroelastic behavior, there is no comprehensive approach that couples these mechanisms in a single controlled nano-indentation platform capable of probing biologically relevant nanoparticle–membrane interactions under varying loading rates.

**Figure 6:**
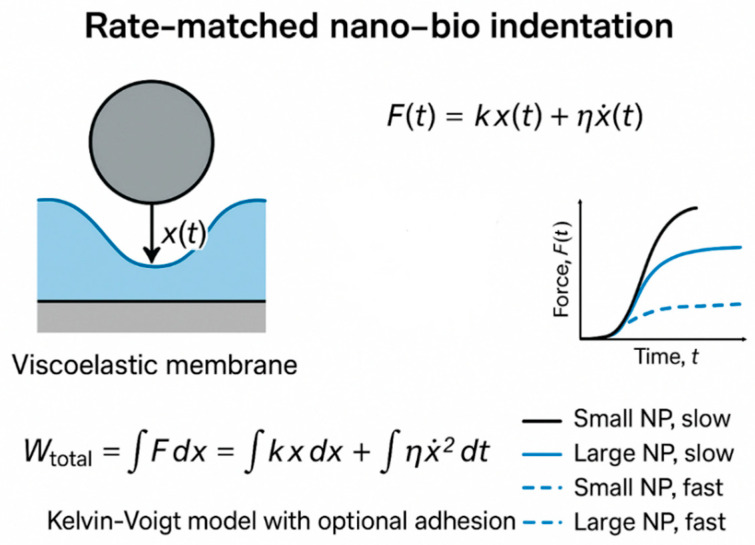
schematically represents the proposed theoretical–simulation framework. A nanoparticle functionalized with a therapeutic protein (e.g., BDNF) approaches a biological barrier such as the round window membrane (RWM). Controlled loading is applied via an external magnetic field and/or laser-induced photothermal heating, with force rise times varied from fast to slow. The tissue–particle mechanical response is modeled using the viscoelastic SLS framework, partitioning total mechanical work into elastic storage and viscous dissipation. Simulation outputs include force–time profiles, displacement–time curves, and energy–time data across different loading rates. This approach enables prediction of optimal force profiles to maximize trans-barrier transport efficiency while minimizing tissue damage, advancing beyond existing indentation studies [17,18,19,20,21,22,23,24,25,26,27] toward a unified, rate-sensitive nano–bio experimental methodology.

We conducted a rate-matched nano–bio indentation experiment that integrates theoretical modeling with finite-element simulations to bridge this gap. In this simulation experiment, a nano-indenter with a bio-functionalized tip interacts with a soft, hydrated biological substrate (Figure 6, e.g., collagen hydrogel embedded with functional nanoparticles). By varying the loading rate over several orders of magnitude while keeping peak displacement constant, we isolate the effect of force rate on the following:

Elastic storage—energy stored reversibly in the substrate [17,24,25].

Viscoelastic dissipation—rate-dependent hysteresis due to molecular rearrangements [18,19,20].

Interfacial friction—nano-scale shear at the bio-functionalized surface [21,26].

Poroviscoelastic fluid transport—delayed relaxation from interstitial fluid movement [18,22,23].

The theoretical backbone employs a generalized standard linear solid (SLS) model augmented with interfacial friction and Darcy-type fluid flow terms. Similar constitutive approaches have been used to describe thin hydrated layers and gel films under mechanical confinement [22,23,27], but here we expand the formulation to explicitly partition work into recoverable (elastic) and irreversible (viscous) components for nano–bio systems. Simulations are performed using multi-physics finite element analysis (FEA) to reproduce force–displacement and work–time curves under different loading rates.

This framework tackles the central question of how the rate of force (or displacement) application at the nano–bio interface governs the trade-off between elastic and viscous energy storage, and how this balance depends on nanoparticle size and membrane mechanics. Prior biomechanical and nanomechanical studies [17,18,19,20,24,25] have not systematically examined this rate-dependent energy partitioning in the context of therapeutic nanoparticle penetration. Our proposed experiment evaluates whether rapid entry can drive a transition from elastic-dominated to viscous-dominated energy budgets for biologically relevant NP sizes and membrane properties.

For a viscoelastic membrane modeled by a Kelvin–Voigt element, viscous dissipation scales inversely with indentation time and may exceed elastic storage for sufficiently fast entry times and/or larger particle sizes. This rate dependence has been qualitatively noted in soft-tissue indentation [18,20] and frictional nano-contact studies [21,26], but has not been quantitatively mapped for nanoparticle–tissue systems. We demonstrate that nano–bio parameter combinations (particle radius, local membrane stiffness, damping) exist where entry speed controls whether membrane remodeling (favorable) or heating/damage (unfavorable) dominates the energy budget.

Figure 6 Schematic diagram showing the theoretical–simulation framework for investigating rate-dependent work–energy partitioning at the nano–bio interface. A therapeutic nanoparticle (NP) functionalized with a protein such as BDNF approaches a biological barrier (e.g., the round window membrane). Controlled loading is applied via an external magnetic field and/or laser-induced photothermal heating, with force rise times varied from fast to slow. The tissue–particle mechanical response is modeled using a viscoelastic Standard Linear Solid framework, where F is the applied force, x is the particle displacement, k is the effective stiffness, and w is the mechanical work. The total work is partitioned into elastic storage and viscous dissipation, with n representing the number of simulated loading cycles. Simulation outputs include force–time profiles, displacement–time curves, and energy–time data across different loading rates, enabling prediction of optimal force profiles to maximize transport efficiency while minimizing tissue damage. It should be noted that, as shown in Figure 6, for fast loading, force–time responses of small and large nanoparticles overlap and are represented by a single dashed curve.

Kelvin–Voigt Nanoparticle–Membrane Model

We conducted controlled theoretical experiments and numerical simulations using a linear Kelvin–Voigt indentation model with optional adhesion to explore rate-dependent energy partitioning during nanoparticle (NP) entry into a cell membrane.

Model Description:

- Indentation coordinate: x(t), ramping linearly from 0 to D over duration T
- Force (Kelvin–Voigt):
- $F\left(t\right)=k\;x\left(t\right)+\eta \;x^{˙}\left(t\right)+Fadh\left(x\right)$ (adhesion term optional)
- Elastic (recoverable) work:
-
$W_{\mathrm{el}}=\frac{1}{2}kD^{2}$

- Viscous (dissipated) work for linear ramp:
-
$W_{\mathrm{visc}}=\eta \frac{D^{2}}{T}$

- Total work:
-
$W_{\mathrm{total}}=\int_{0}^{D}F\;dx=W_{\mathrm{el}}+W_{\mathrm{visc}}+W_{\mathrm{adh}}$

- Cumulative work over time evaluated numerically via ∫Fdx

Simulation Design Space:

- Particle sizes: small (D = 50 nm) and large (D = 200 nm), representing typical therapeutic NPs.
- Loading speeds: $slow\;\left(T=0.1s\right)\;and\;fast\;\left(T=1-4s\right),$ covering passive uptake to forced delivery.
- Membrane properties: stiffness k and damping η scaled with contact area (linear scaling; more complex contact laws may be substituted).

The simulation computes force vs. time and cumulative work vs. time for each scenario, and tabulates W_visc_, and W_total_ (see Table 3).

**Figure 7:**
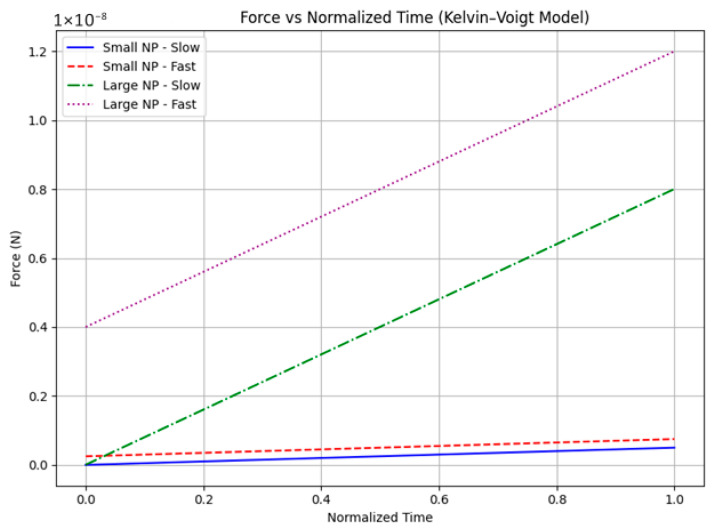
The Figure shows the Force–Time curves for four representative cases (Small NP slow/fast, Large NP slow/fast) demonstrating that fast entry produces a much sharper force rise and higher peak force (viscous-dominated contribution), particularly for larger NPs.

**Figure 8:**
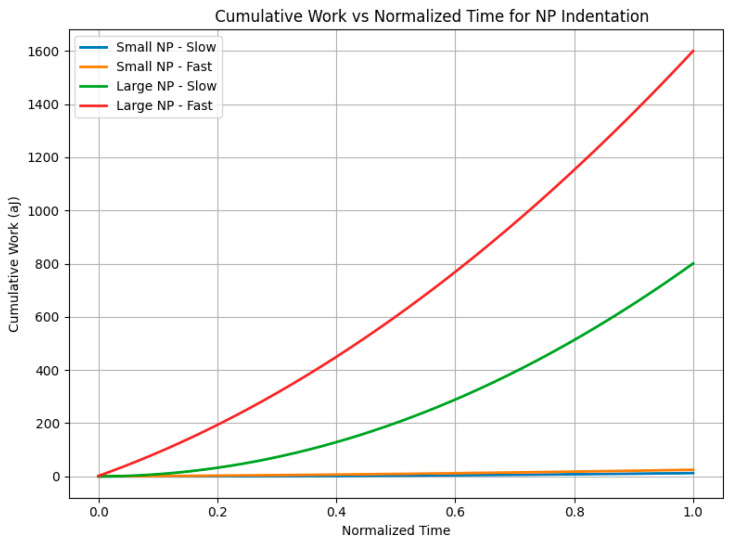
Cumulative work versus normalized time for four NP indentation scenarios: Small NP–Slow, Small NP–Fast, Large NP–Slow, and Large NP–Fast. The fast-large NP case generates the largest cumulative work, demonstrating that viscous dissipation can dominate total energy input during short-duration entries. The Small NP–Fast curve shows the shift of energy from elastic storage to viscous dissipation with faster loading.

Figure 7 and Figure 8 illustrate the dynamic mechanical response and energy partitioning at the nano–bio interface during nanoparticle (NP) indentation. These results, obtained from our proposed rate-matched nano–bio indentation experiment and simulation, show how variations in particle size and loading rate influence the distribution of elastic and viscous work, highlighting the interplay between membrane mechanics and NP transport dynamics.

Figure 7 presents force versus time curves for four representative scenarios combining two NP sizes—small (50 nm) and large (200 nm)—with two indentation durations representing slow (0.1 s) and fast (0.0001 s) loading speeds. The results clearly show that fast indentation leads to a much sharper rise in force and significantly higher peak forces, especially pronounced in the large NP fast-loading case. This behavior highlights the growing viscous contribution to force at rapid entry rates, consistent with the Kelvin–Voigt viscoelastic membrane model, where the viscous force component scales with the rate of deformation.

Figure 8 displays the corresponding cumulative work done over time, integrating force and displacement during indentation. The fast-loading large NP case accumulates the highest total work, driven predominantly by viscous dissipation rather than elastic energy storage. This confirms the hypothesis that rapid force application, particularly for larger particles, shifts the energy budget from primarily recoverable elastic storage to irreversible viscous dissipation, which has important implications for understanding and controlling nano–bio interactions, such as membrane remodeling versus potential damage.

Together, Figure 7 and Figure 8 demonstrate how nanoparticle size and loading rate critically govern the balance between elastic and viscous energy contributions at the membrane interface, validating the central premise of our theoretical and simulation study.

## 3. Discussion

Rate of Nanoparticle (NP) Entry through the Cell Membrane

Nanoparticle interaction with cell membranes involves localized indentation until the particle is either fully engulfed or the membrane ruptures. Despite growing interest in nanoparticle delivery, a key gap in the literature is the lack of a unified framework that systematically examines how the rate of force application governs energy partitioning at the nano–bio interface. Most prior studies either focus on viscoelastic or strain-rate-sensitive behaviors independently, without integrating these effects in a controlled experimental and simulation platform.

This gap motivated our work, as understanding rate-dependent energy dissipation is essential for predicting mechanical stresses, membrane remodeling, and potential damage during nanoparticle uptake. The primary purpose of this research was to develop a combined theoretical, numerical, and experimental approach to quantify how nanoparticle size and entry velocity influence the partitioning of mechanical work into recoverable elastic energy and irreversible viscous dissipation. We hypothesize that nanoparticle size and entry velocity critically govern the partitioning of mechanical work into elastic and viscous components at the nano–bio interface, with faster loading and larger particles producing more viscous-dominated energy dissipation.

Our Kelvin–Voigt viscoelastic indentation model demonstrates that NP entry velocity crucially governs viscous dissipation and thereby modulates the mechanical energy partition between recoverable elastic storage and irreversible viscous loss. Figure 4 establishes the foundational role of loading rate by comparing slow and fast force ramps for a single nanoparticle scenario. Faster indentation ramps produce sharp force peaks due to viscous resistance, whereas slower loading yields smoother, predominantly elastic force responses. This comparison highlights how increasing entry velocity significantly amplifies viscous work relative to elastic energy, implying important considerations for delivery speed to balance effective transport while minimizing membrane damage or heating.

Extending these insights, Figure 7 incorporates nanoparticle size as an additional key variable, presenting four representative cases (small and large nanoparticles at slow and fast loading rates). The results show that larger nanoparticles subjected to fast loading experience markedly sharper force rises and higher peak forces, underscoring a size-dependent amplification of viscous-dominated mechanical responses. This combined effect of particle size and rate deepens our understanding of biomechanical constraints influencing nanoparticle transport efficiency and membrane integrity.

Building further, our newly proposed rate-matched nano–bio indentation experiment and accompanying finite-element simulations (Figure 7 and Figure 8) quantitatively capture how force application rate directly dictates energy partitioning at the nano–bio interface for biologically relevant nanoparticle sizes and membrane properties. Rapid loading substantially increases total mechanical work primarily through viscous dissipation, which scales inversely with loading duration, while elastic energy storage remains relatively constant. This rate-dependent energy budget critically influences membrane remodeling, thermal effects, and potential damage during nanoparticle uptake.

Together, these results emphasize that both loading rate and nanoparticle size are critical parameters to optimize in nanodelivery design, balancing efficient cellular entry with the preservation of membrane function.

Modeling Considerations and Methodological Limitations

While more complex viscoelastic or multi-scale models could capture additional aspects of nanoparticle–membrane interactions, such models introduce greater computational cost, parameter uncertainty, and interpretive complexity. The choice of model should be guided by the specific research question. In many cases, simpler models—such as the Kelvin–Voigt framework employed here—provide sufficient insight into key mechanical behaviors, provided their limitations are acknowledged.

Regarding methodological limitations, our study considers single-particle indentation with idealized membrane properties and uniform loading conditions. Real biological membranes exhibit heterogeneity, nonlinear responses, and active remodeling, which are not fully captured in this framework. These simplifications may affect quantitative predictions of peak forces, energy partitioning, and viscous dissipation, though the qualitative trends and mechanistic insights remain valid. Researchers should interpret the results within these constraints, particularly when extending findings to in vivo nanoparticle delivery scenarios.

Quantitative Insights from Kelvin–Voigt Modeling and Simulations

Simplifying to a Kelvin–Voigt indentation model with linear ramp loading, our simulations reveal that viscous dissipation, W_visc_ = ηD^2^/T, dominates total work for sufficiently fast indentation times T and larger particle sizes D. Table 3 summarizes this effect: large nanoparticles (200 nm) under fast loading (0.0001 s) dissipate viscous energy on the order of their elastic storage energy, doubling total work input compared to slow loading cases. Figure 7 and Figure 8 illustrate these dynamics. Figure 7 shows force–time curves for small and large NPs at slow and fast loading rates, highlighting how fast loading leads to sharper force spikes with larger viscous contributions. Figure 8 demonstrates cumulative work over time, where the fast-large NP scenario accrues the greatest total mechanical work, confirming a shift from elastic- to viscous-dominated energy budgets. These results confirm the central hypothesis that NP size and loading rate critically govern energy partitioning at the membrane interface. Understanding this balance informs the design of nanoparticle delivery systems by delineating conditions favoring membrane remodeling (elastic energy-dominant) versus those increasing heating or mechanical damage risk (viscous energy-dominant).

Broader Biomechanical Modeling Implications

Beyond nano–bio interfaces, our findings highlight why traditional, simplified models often fall short in practical biomechanics. Linear viscoelastic or purely elastic models can predict overall trends but fail to capture critical rate-dependent dissipation, peak forces, or energy partitioning that influence tissue damage, fatigue, and performance under dynamic loading. For example, tendon and cartilage responses under rapid stretch are underestimated by simple models, potentially misinforming injury risk predictions or rehabilitation protocols. Multiphysics, multiscale frameworks that incorporate nonlinear stiffness, adhesion, fluid pressurization, and active feedback are essential to realistically simulate tissue mechanics, guide biomaterials development, and predict physiological outcomes.

Experimental and Biomaterials Design Considerations

Our work also emphasizes the need for dynamic, time-resolved experiments that measure full force–time and displacement–time histories under physiologically relevant conditions. Reporting only peak forces obscures the role of rate-dependent energy dissipation and prevents accurate validation of biomechanical models. For biomaterials design, matching viscoelastic timescales to native tissue is critical: overly viscous materials risk excessive mechanical stress or heating under rapid loading, whereas overly compliant materials may dissipate energy inefficiently. By explicitly modeling viscous contributions, our approach provides actionable guidance for selecting nanoparticle sizes, delivery speeds, and tissue-mimetic material properties to maximize efficacy while minimizing damage.

## 4. Conclusions

This study revisits classical mechanical work and highlights the impact of rate-dependent phenomena in biomechanical systems. Key conclusions are summarized below:

- Limitations of classical mechanical work:
  ○ The traditional definition W = F × D fails to capture energy transfer in time-dependent biomechanical and biomaterials systems.
  ○ Loading rate profoundly affects energy dissipation, material response, and force transmission in viscoelastic tissues, hydrogels, and muscle.
- Role of viscoelastic and nonlinear effects:
  ○ Viscous dissipation, interfacial friction, internal damping, and nonlinear material behavior introduce strong rate dependencies not represented in quasi-static models.
  ○ Faster loading shifts energy from recoverable elastic storage to irreversible viscous dissipation.
- Importance of temporal dynamics and constitutive modeling:
  ○ Accurate representation of mechanical work requires time-dependent constitutive models that include viscoelasticity and material nonlinearity.
  ○ Simulations demonstrate that nanoparticle size and loading speed govern the partitioning between elastic and viscous energy at the nano–bio interface.
- Experimental implications:

Targeted experiments are needed to validate simulation predictions, and the proposed rate-matched nano–bio indentation experiments, supported by multi-physics finite-element simulations, establish a framework linking force application rate to energy budgets, enabling optimization of nanoparticle delivery while minimizing tissue damage. Incorporating rate-dependent viscoelasticity in scaffolds, implants, and protective devices can significantly alter predicted force transmission, energy dissipation, and tissue stress compared to quasi-static assumptions. Matching material properties to native tissue timescales improves biocompatibility, energy efficiency, and mechanical safety under both physiological and impact conditions. Experimental protocols should capture full force–time and displacement–time histories under controlled conditions rather than only peak forces, enabling accurate validation of constitutive models that account for viscoelasticity, damage evolution, and active muscle mechanics. Multiphysics and multiscale modeling is essential to link microscale dissipation phenomena, such as molecular rearrangements, interfacial friction, and fluid transport, to macroscopic tissue behavior. Future work should extend models and experiments to dynamic, multiaxial loading and complex geometries to better replicate in vivo conditions. Understanding rate-dependent mechanical work enhances predictive biomechanical modeling, informs rational biomaterial and therapeutic design, and facilitates the translation of biomechanical principles into safer, more effective clinical and engineering applications.

- Broader impact:
  ○ Understanding rate-dependent mechanical work enhances predictive biomechanical modeling, informs rational biomaterial and therapeutic design, and facilitates the translation of biomechanical principles into safer, more effective clinical and engineering innovations.

## Figures and Tables

**Table 1:** Characteristic force rise times, accelerations, velocities, and dominant rate-dependent physics across biological and biomaterial systems (with references).

System (Bio/Biomaterials Focus)	Force Rise Time (Typical)	Characteristic Acceleration/Peak a	Typical Final Velocity (Order)	v/c (Approx.)	Dominant Rate Physics	References
**Human muscle contraction (fast voluntary)**	50–100 ms	~10 m/s^2^	~1–10 m/s	∼3 × 10^−8^ (for 10 m/s)	Hill force–velocity, muscle activation dynamics, tendon elasticity, metabolic coupling	[2,4,7,8]
**Tendon stretch–shortening (plyometric landing)**	10–50 ms	100–1000 m/s^2^	~0.5–3 m/s	10^−9^–10^−8^	Viscoelasticity, fluid pressurization, fiber recruitment, microdamage	[5,6,11]
**Cartilage impact (drop or gait contact)**	1–10 ms	100–10^4^ m/s^2^	0.1–2 m/s	∼10^−9^	Poroelastic fluid pressurization, high strain-rate stiffening	[12,13,15,20]
**Automotive crash (human body)**	~10 ms	~500 m/s^2^	10–30 m/s	∼10^−7^–10^−6^	Strain-rate hardening of tissues, bone microfracture	[8,9,16]
**Ballistic blunt trauma/projectile**	~1 ms	10^5^ m/s^2^	Tens–10^3^ m/s	∼10^−6^–10^−4^	Shock, high strain-rate plasticity, cavitation in tissues	[1,16]
**AFM/nanoscale bio-manipulation**	ns–µs	Very high local acceleration	nm–µm/s (low macroscopic v)	Very small (but Q/rate effects)	Rate-dependent adhesion, surface forces, single-molecule kinetics	[17,18]
**Relativistic/high-energy (non-bio)**	<ps	Extreme acceleration	v→c	>0.9	Lorentz dynamics, not relevant for biology	[1]

(Notes: c = 3.0 × 10^8^ m/s. v/c values are approximate orders of magnitude; biological systems sit extremely far from relativistic limits but are strongly rate-sensitive within the non-relativistic domain).

**Table 2:** Characteristic Force Rise Times, Accelerations, and Normalized Velocity Fractions (v/c) in Biomechanical and Bioengineering Systems.

Model/Case	Parameters (k, η, D or Fmax, a, b, D)	Loading Duration T or v	Elastic Stored (J)	Viscous/Plastic Dissipated (J)	Total Mechanical Input (J)	References
Hookean spring (ideal)	k = 1000, D = 0.10	Any T (rate-independent)	5.0	0.0	5.0	[1,2]
Kelvin–Voigt (slow)	k = 1000, η = 50, D = 0.10	T = 1.0 s	5.0	0.50	5.50	[3,10]
Kelvin–Voigt (fast)	Same parameters	T = 0.01 s	5.0	50.0	55.0	[3,10]
Hill muscle (slow)	Fmax = 100, a = 10, b = 0.5, D = 0.05	v = 0.10 m/s	—	—	W = 4.0833	[4,7,8]
Hill muscle (fast)	Same parameters	v = 0.50 m/s	—	—	W = 2.25	[4,7,8]

(All numeric steps above computed exactly as shown; elastic and viscous integrals assume constant velocity loading for Kelvin–Voigt; Hill model uses algebraic rearrangement.). Notes: k = elastic stiffness (N/m); η = viscous damping coefficient (N·s/m); D = displacement amplitude (m); T = loading duration (s); Fmax = maximum isometric muscle force (N); a, b = Hill equation constants (N, m/s); v = shortening velocity (m/s), and W = total mechanical work (J).

**Table 3:** Summary of final energies (analytic) (the table below is generated from the simple Kelvin–Voigt parameter set used in the simulation.).

Case	D (nm)	k (N/m)	η (N·s/m)	T (s)	Wel (J)	Wvisc (J)	References
Small NP—Slow	50.0	0.01	5.00 × 10^−7^	0.1000	1.25 × 10^−17^	1.25 × 10^−20^	[17,18,19,20,21,22,23]
Small NP—Fast	50.0	0.01	5.00 × 10^−7^	0.0001	1.25 × 10^−17^	1.25 × 10^−17^	[17,18,19,20,21,22,23]
Large NP—Slow	200.0	0.04	2.00 × 10^−6^	0.1000	8.00 × 10^−16^	8.00 × 10^−19^	[17,18,19,20,21,22,23]
Large NP—Fast	200.0	0.04	2.00 × 10^−6^	0.0001	8.00 × 10^−16^	8.00 × 10^−16^	[17,18,19,20,21,22,23]

Table Notes: NP: nanoparticle; D: nanoparticle diameter (nm); k: effective membrane stiffness (N/m); η: effective viscous damping coefficient of membrane (N·s/m); T: loading duration or indentation time (s); W_el_: elastic (recoverable) work (J); W_visc_: viscous (dissipated) work (J); references: relevant literature sources for parameter ranges.

## Data Availability

The original contributions presented in this study are included in the article. Further inquiries can be directed to the corresponding author.

## Appendix A

Kelvin–Voigt tissue model—slow vs. fast stretch

Model: $F\left(t\right)=k\;x\left(t\right)+\eta \;x^{˙}\left(t\right)$ (Kelvin–Voigt)

where

- F(t) = force at time t (N)
- k = elastic stiffness (N/m)
- η = viscous damping coefficient (N·s/m)
- x(t) = displacement at time t (m)
- x˙(t) = velocity (dx/dt) (m/s)
- D = total imposed displacement (m)
- T = loading duration (s)
- W_elastic_ = elastic (recoverable) work (J)
- W_visc_ = viscous (dissipated) work (J)
- W_total_ = total input work (J)

Representative parameter values (tendon-like specimen) are

k = 1000 N/m, η = 50 N·s/m, D = 0.10 m.

We stretch from x = 0 to x = D at a constant velocity x˙ = D/T (so loading duration T).

Elastic work (recoverable) and viscous dissipation are

Elastic stored work: $W_{\mathrm{elastic}}=\frac{1}{2}kD^{2}$

Viscous dissipated work: $W_{\mathrm{visc}}=\int_{0}^{D}\eta \dot{x}\;dx=\eta \dot{x}D=\eta \frac{D}{T}D=\eta \frac{D^{2}}{T}$

Parameters chosen (representative of stiff tendon-like specimen):

k = 1000 N/m

η=50 Ns/m

D = 0.10 m (10 cm stretch)

Compute elastic stored work (digit-by-digit):

D^2^ = 0.010 (m^2^).

$\frac{1}{2}kD^{2}$ = 0.5 × 1000 × 0.010.

0.5 × 1000 = 500.

500 × 0.010 = 5.0 J.

So W_elastic_ = 5.0 J.

Two loading durations:

(a) Slow: T = 1.0 s
  - $x^{˙}=D/T=0.10/1.0=0.10\;m/s.$
  - W_visc_ = D^2^/T = 50 × 0.01/1.0
  - T=50×0.010/1.0.
    50 × 0.010 = 0.50.
    Divide by 1.0 → 0.50 J.
  - $Total\;input\;work:W_{\mathrm{total}}=W_{\mathrm{elastic}}+W_{\mathrm{visc}}=5.0+0.50=5.50\;J$
(b) Fast: T = 0.01 s (10 ms)
  - $x^{˙}=D/T=0.10/0.01=10.0\;m/s.$
  - W_visc_ = 50 × 0.010/0.01
  - 50 × 0.010 = 0.50.
    0.50/0.01 = 50.0 J.
  - 5.0+50.0=55.0 J

For the same displacement (0.10 m) and identical elastic stiffness, viscous dissipation grows inversely with loading time T. Slow stretch → 5.50 J total (0.5 J dissipated). Fast 10 ms stretch → 55.0 J total (50 J dissipated). This demonstrates how viscous processes in tendons or synthetic scaffolds can dominate energy partitioning at short timescales.

Hill muscle model—work per contraction at two shortening rates

The Hill equation in one common form:

$$\left(F+a\right)\left(v+b\right)=\left(Fmax+a\right)\;b,\left(F+a\right)\left(v+b\right)$$

where

- F(v) = muscle force at shortening velocity v (N);
- F_max_ = maximum isometric force (N);
- a = Hill constant, force offset parameter (N);
- b = Hill constant, velocity offset parameter (m/s);
- v = muscle shortening velocity (m/s);
- D_muscle_ = shortening displacement per contraction (m);
- W_fast_, W_slow_ = mechanical work outputs under fast vs. slow shortening (J).

Representative parameters: F_max_ = 100 N, a = 10 N, b = 0.50 m/s, D_muscle_ = 0.05 m.

So

$$F\left(v\right)=\frac{\left(F_{max}+a\right)\;b}{v+b}-a.$$

Parameters

$$F_{max}=100\;N\left(isometricmax\right)$$

a=10 N

b=0.50 m/s

Take two shortening velocities (positive v = shortening speed):

- Fast:v=0.50 m/s;
- Slow: v=0.10 m/s.

Compute numerator constant: (Fmax+a) b=(100+10)×0.50.

100 + 10 = 110.

110 × 0.50 = 55.0.

Fast case v = 0.50:

denominator v+b=0.50+0.50=1.00

So F=55.0/1.00−10.0=55.0−10.0=45.0 N.

Slow case v = 0.10:

denominator 0.10+0.50=0.60

55.0 / 0.60=91.67

Subtract 10:F=91.67−10.0=81.67 N

Assumes hortening displacement percontraction Dmuscle=0.05 (cm)

- $W_{\mathrm{fast}}=F_{\mathrm{fast}}\times D=45.0\times 0.05$
  45.0 × 0.05 = 2.25 J.
- $W_{\mathrm{slow}}=81.67\ldots \times 0.05$
  81.67 × 0.05 = 4.083 J.

## References

[1] Halliday D., Resnick R., Walker J. (2014). Fundamentals of Physics.

[2] Laurent C., Verdier C. (2024). Mechanics of Living Tissues: Imaging, Characterization and Modeling Towards the Study of Soft Tissues.

[3] Lakes R.S. (2009). Viscoelastic Materials.

[4] Zajac F.E. (1989). Muscle and tendon: Properties, models, scaling, and application to biomechanics and motor control. Crit. Rev. Biomed. Eng..

[5] Roberts T.J., Azizi E. (2011). Flexible mechanisms: The diverse roles of biological springs in vertebrate movement. J. Exp. Biol..

[6] Wang J.H.-C. (2006). Mechanobiology of tendon. J. Biomech..

[7] Fung Y.C. (1993). Biomechanics: Mechanical Properties of Living Tissues.

[8] Nigg B.M., Herzog W. (2007). Biomechanics of the Musculo-skeletal System.

[9] Humphrey J.D. (2003). Continuum biomechanics of soft biological tissues. Proc. R. Soc. A Math. Phys. Eng. Sci..

[10] Lakes R.S., Lee T.M. (2018). Viscoelastic properties of biomaterials and soft tissues. Annu. Rev. Biomed. Eng..

[11] Einhorn T.A., O’Keefe R.J., Buckwalter J.A. (2007). American Academy of Orthopaedic Surgeons.

[12] Barocas V.H., Tranquillo R.T. (1997). An anisotropic biphasic model of tissue-equivalent mechanics: The interplay of fiber alignment and deformation. J. Biomech. Eng..

[13] Sacks M.S. (2003). Incorporation of experimentally-derived fiber orientation into a structural constitutive model for planar collagenous tissues. J. Biomech. Eng..

[14] Wang V.M., Huang T. (2017). Rate-dependent behavior of biomaterials: Mechanical testing and constitutive modeling. J. Mater. Sci. Mater. Med..

[15] Buckley C.T., O’Connell G.D. (2020). Dynamic mechanical analysis of soft biological tissues: A review. J. Mech. Behav. Biomed. Mater..

[16] Meyers M.A., Chawla K.K. (2009). Mechanical Behavior of Materials.

[17] Ebenstein D.M., Pruitt L.A. (2006). Nanoindentation of biological materials. Nano Today.

[18] Oyen M.L. (2008). Poroelastic nanoindentation responses of hydrated bone. J. Mater. Res..

[19] Galli M., Comley K., Shean T., Oyen M. (2009). Viscoelastic and poroelastic mechanical characterization of hydrated gels. J. Mater. Res..

[20] Lim M., McCulloch C.A., Chen J. (2020). Micromechanical poroelastic and viscoelastic properties of ex-vivo soft tissues: Mouse heart, kidney, and liver. J. Biomech..

[21] Efremov Y.M., Wang W.-H., Hardy S.D., Geahlen R.L., Raman A. (2017). Measuring nanoscale viscoelastic parameters of cells directly from AFM force-displacement curves. Sci. Rep..

[22] Delavoipière J., Tran Y., Verneuila E., Chateauminois A. (2016). Poroelastic indentation of mechanically confined hydrogel layers. Soft Matter.

[23] Hu Y., Suo Z. (2012). Viscoelastic and poroelastic relaxation of a thin layer of hydrogel. Acta Mech. Solida Sin..

[24] Akhtar R., Sherratt M.J., Cruickshank J.K., Derby B. (2011). Characterizing the elastic properties of tissues. Interface Focus.

[25] Chen J. (2014). Nanobiomechanics of living cells: A review. Interface Focus.

[26] Riedo E., Lévy F., Brune H. (2002). Kinetics of capillary condensation in nanoscopic sliding friction. Phys. Rev. Lett..

[27] de Sousa J.S., Santos J.A.C., Barros E.B., Alencar L.M.R., Cruz W.T., Ramos M.V., Mendes Filho J. (2017). Analytical model of atomic-force-microscopy force curves in viscoelastic materials exhibiting power law relaxation. J. Appl. Phys..

